# The Identification of Risk Factors in the Development of Wound Healing Complications in Soft Tissue Sarcoma Patients

**DOI:** 10.1111/iwj.71020

**Published:** 2026-08-27

**Authors:** Luisa Kriens, Jendrik Hardes, Wiebke Guder, Nina Engel, Marcel Dudda, Arne Streitbuerger

**Affiliations:** ^1^ University Hospital Essen Essen Germany

**Keywords:** multimodal therapy, single center risk factors, soft tissue sarcoma, wound healing

## Abstract

Wound‐healing complications (WHCs) remain a significant challenge in soft tissue sarcoma (STS) surgery, contributing to increased morbidity, prolonged hospitalisation, and impaired functional outcomes. This study aimed to identify predictors of WHC development in STS patients to improve therapeutic strategies and patient recovery. We conducted a single‐institution, retrospective analysis of 318 STS patients treated between April 2019 and June 2021. Variables assessed included demographic data, tumour characteristics, treatment modalities, surgical interventions, and follow‐up outcomes. Statistical analyses used chi‐square tests, Kaplan–Meier estimates, and multivariate regression. WHCs occurred in 30.8% of patients. Significant negative predictors included high tumour grade (*p* = 0.018), presence of metastasis (*p* < 0.001), bone tissue resection (*p* = 0.005), number of revision procedures (*p* < 0.001), secondary wound closure (*p* < 0.001), and tumour relapse frequency (*p* = 0.012). WHCs were also associated with increased risk of death (*p* = 0.004) and longer initial hospitalisation (*p* < 0.001). Chemotherapy showed a protective effect when stratified by tumour grade. Patients with high‐grade tumours and multiple surgical interventions represent a high‐risk group for WHC development. These findings highlight the importance of ongoing treatment reevaluation and suggest that chemotherapy may offer beneficial outcomes in selected cases.

## Key Points

- The number of local surgical interventions increases the risk of WHC.
- Primary wound closure is less susceptible to WHC.
- When susceptible, early chemotherapy shows a protective effect against WHC development.
- Early transfer to a specialized center can provide early specialized assessment and therapy planning to provide optimal treatment.

## 1 Introduction

In sarcoma surgery, postoperative wound‐healing complications (WHC) are a common concern. Because soft‐tissue sarcomas (STS) account for only 1% of oncologic diseases, most WHC studies are small, with up to 200 participants. WHC occur in 30%–40% of cases and are associated with increased readmissions, reoperations, poorer functional outcomes, and limb amputations [1, 2, 3, 4, 5, 6]. Known risk factors include infection, necrosis, hematoma, and seroma, with incidence rates ranging from 16% to 53%. Research repeatedly shows that age, tumour size, and location are key risk factors. Other variables include diabetes mellitus, (neo‐)adjuvant radiotherapy and chemotherapy, and initial wound‐closure methods such as negative‐pressure wound therapy (NPWT), muscle flap transplantation, or skin grafts [7, 8, 9, 10, 11, 12]. To better understand how these diverse factors affect outcomes, our study analysed individual risk factors and their interactions. The main treatment for STS involves a multidisciplinary approach that combines surgery and often radiotherapy. Many studies have explored the impact of radiotherapy on WHC development, focusing on neoadjuvant, intraoperative, or adjuvant use. Despite ongoing high rates of WHC, further research is necessary to assess the effects of modern therapies and to compare them directly to improve patient outcomes. We studied a diverse cohort of 22 tumour types across various stages, all treated at our large superregional sarcoma centre. To minimise bias from treatment errors or delays, we included only patients diagnosed and managed at our facility in accordance with standardised protocols. Our goal was to analyse multiple factors and identify interactions that influence wound healing in STS treatment.

## 2 Methods

### 2.1 Patients

This was a single‐institutional retrospective study conducted over 2 years from April 2019 to June 2021. In total, 318 patients were included. They had to be initially diagnosed and at least once treated and discussed by the tumour board at our centre during the prior two‐year period mentioned above.

### 2.2 Data Collection and Definitions

The Ethics Committee approved the study before initiation. For this retrospective research, all STS cases diagnosed and treated between April 2019 and June 2021 were initially selected. We gathered basic medical data such as age, gender, and diagnosis date. The analysis further explored tumour characteristics and treatment modalities, including chemo‐, radio‐, and combined therapies. The cohort consisted of 178 males and 140 females, with a median age of 64.36 years (range 2–96 years). No patients were lost to follow‐up. Twenty‐seven patients died during the observed period. These patients were still included because they had completed treatment and had at least one follow‐up visit at our facility. Follow‐up was documented from the date of diagnosis through the last recorded appointment, including after the specified time period. The cause of death, whether oncologic or not, was not further investigated in this study. Tumours were classified as high‐grade (G2 and G3) or low‐grade (G1). Relapses and revision surgeries were documented from the initial diagnosis, regardless of whether treatment was provided in specialised or nonspecialised centres. Relapse was defined as any local recurrence after treatment. Revision procedures included any surgeries performed after the primary operation, with documented R0 or R1 status. This included surgeries for margin control after incomplete resections, as well as surgeries during WHC treatment. Prolonged wound healing was defined as a duration exceeding 3 weeks and/or the use of VAC therapy. Overall, 30.8% (*n* = 98) of patients experienced at least one WHC, defined as wound infection, dehiscence, or delayed healing, with or without additional surgery. Analyses were conducted using Chi‐Square, Kaplan–Meier, and *t*‐tests in SPSS Version 29.0.0.0 and Microsoft Excel. Graphics and tables were produced using SPSS or LaTeX. Our focus was on identifying potential predictors, whether alone or in combination, that could influence outcomes positively or negatively.

## 3 Results

### 3.1 General Clinical Variables

Beginning with a general overview, Table 1 summarizes all categories viewed that apply to general clinical information for our viewed collective. Identified significant parameters were grading (*p* = 0.018; OR 0.556), the presence of metastasis (*p <* 0.001; OR 2547), the length of stay after the first surgery (*p <* 0.001; OR 11.75), and the event of death (*p* = 0.004; OR 1.4–6.9). The median estimated age of the cohort was 64.36 years [2–96]. Patients with a diagnosed WHC event tended to be about 4016 (*p* = 0.228; CI −0.4 to 8.4), with an average age of 67.14 years [25–96], compared to patients not affected by WHC. Non‐affected patients presented an average age of 63.13 years [2–95] (*p* = 0.038).

**TABLE 1:** Association between clinical variables and wound healing complications.

Category	Subgroup	WHC		No‐WHC		Total		*p*	Odds ratio (CI)
		Count	%	Count	%	Count	%		
Diabetes mellitus	Yes	11	3.5	17	5.3	28	8.8	0.310	
	No	87	27.4	203	63.8	290	91.2		
Sex	Male	59	18.6	119	37.4	178	56.0	0.311	
	Female	39	12.3	101	31.8	140	44.0		
Grading	Low grade	35	11.0	110	34.6	145	45.6	**0.018**	0.556 (0.3–0.9)
	High grade	63	19.8	110	34.6	173	54.4		
Size (cm)		13.95		11.95		12.56		0.199	
Size (*<*/*>* 11 cm)	< 11.00 cm	39	12.9	110	36.4	149	49.3	0.139	
	> 11.00 cm	52	17.2	101	33.4	153	50.7		
Localisation	Upper arm (UA)	5	1.6	13	4.1	18	5.7	0.505	
	Lower arm (LA)	2	0.6	14	4.4	16	5.0		
	Upper leg (UL)	54	17.0	117	36.9	171	53.9		
	Lower leg (LL)	13	4.1	20	6.3	33	10.4		
	Spine (Sp)	1	0.3	5	1.6	6	1.9		
	Pelvis (Pe)	12	3.8	20	6.3	32	10.1		
	Other (Oth)	11	3.5	30	9.5	41	12.9		
Metastasis	Yes	45	14.2	55	17.3	100	31.4	**< 0.001**	2.547 (1.5–4.2)
	No	53	16.7	165	51.9	218	68.6		
Further oncologic disease	Yes	13	4.1	22	6.9	35	11.0		0.390
	No	85	26.7	198	62.3	283	89.0		
Length of stay (d)		22.11		10.36		13.85		**< 0.001**	11.750 (8.6–14.9)
Follow up (d)		958.16		826.26		867.80		0.520	
Follow up (mth)		30.99		26.67		28.03		0.532	
Death	Yes	15	4.7	12	3.8	27	8.5	**0.005**	3.029 (1.4–6.7)
	No	85	26.7	206	64.8	291	91.5		
Death (d)		1134.33		1564.75		1325.63		0.481	
Death (mth)		36.80		50.92		43.07		0.481	
Age (y)		67.14		63.13		64.36		0.228	

*Note:* d, days; mth, months; y, years. Bold values indicate significant *p*‐values.

No patients were lost to FU. The length of follow‐up had no significant impact (FU (d): *p* = 0.520; FU (month): *p* = 0.532). Twenty‐seven patients (8.5%) died during the study period; 15 (4.7%) had a diagnosed WHC. Data analysis indicates that patients with a WHC diagnosis have a 3.029 times higher risk of death (*p* = 0.005; OR 3.029).

Analysis of clinical data on tumour disease revealed that the presence of metastasis significantly impacts WHC development. Patients with metastatic tumour disease exhibited a 2.5‐fold higher incidence of WHC compared with those without metastatic involvement (*p <* 0.001; OR 2.547; CI 1.54.2). The timing of appearance, whether at diagnosis or after initial surgery, did not significantly influence the outcome. In contrast, the presence of a further oncologic disease (FOD) showed no significant association with WHC development (*p* = 0.312).

When examining specific tumour characteristics, tumour localisation surprisingly was not a significant factor (*p* = 0.505). Similarly, tumour size, whether measured in metric or nominal units, did not differ significantly (*p* = 0.199/*p* = 0.139).

Tumour grading at initial diagnosis was highly significantly predictive. Specifically, low‐grade tumours were associated with a substantially lower likelihood of WHC development, with an odds ratio of 0.556, indicating a 44.4% reduction in risk compared to high‐grade tumours. (*p* = 0.018; OR 0.556; CI 0.3–0.9).

Gender and diabetes mellitus did not appear to influence WHC development, with *p* values of 0.311 and 0.310, respectively. The most frequent histologic subtypes included undifferentiated pleomorphic sarcoma (33.3%), myxoid‐like sarcoma (myxoid fibrosarcoma (12.9%), myxoid liposarcoma (10.6%)), and liposarcoma (10.9%).

### 3.2 Surgical Variables

Table 2 summarizes all variables analysed that may be associated with surgical factors influencing WHC development. Interestingly, 5 out of 8 categories implied a significant influence on WHC development. Chi‐square analysis shows a 2.4‐fold increase in the risk of WHC development in patients with bone tissue involvement, with removal of affected bone tissue (*p* = 0.005; OR 2.294; CI 1.3–4.1). 86.7% of patients who had bone tissue surgically removed developed a WHC during their course of treatment.

**TABLE 2:** Association between surgical variables and wound healing complications.

Category	Subgroup	WHC		No‐WHC		Total		*p*	Odds ratio (CI)
		Count	%	Count	%	Count	%		
Bone tissue resection	Yes	27	8.5	31	9.8	58	18.4	**0.005**	2.294 (1.3‐4.1)
	No	71	22.5	187	59.2	258	81.6		
R‐Status	0	75	23.6	172	54.1	247	77.7	0.113	
	1	16	5.0	31	9.7	47	14.8		
	2	5	1.6	2	0.6	7	2.2		
	3	4	1.3	13	4.1	17	5.3		
Revision procedures	Yes	38	11.9	41	12.9	79	24.8	**< 0.001**	2.765 (1.6‐4.7)
	No	60	18.9	179	56.3	239	75.2		
Number of revisions		0.73		0.26		0.41		**< 0.001**	(0.3–0.8)
Wound closure	Primary	74	23.4	203	64.2	277	87.7	**< 0.001**	0.228 (0.1–0.5)
	Secondary	24	7.6	15	4.7	39	12.3		
Amputation	Yes	5	5.1	1	1.0	6	6.1	0.108	
	No	89	90.8	3	3.1	92	93.9		
Infection	Yes	53	53.5	0	0	53	53.5	0.028^a^	
	No	42	42.4	4	4	46	46.5		
Relapses	0	70	22.1	187	59.0	257	81.1	**0.012**	0.575 (0.4‐0.8)
	1	15	4.7	23	7.3	38	12.0		
	2	8	2.5	6	1.9	14	4.4		
	3	3	0.9	3	0.9	6	1.9		
	4	2	0.6	0	0	2	0.6		

*Note:* Bold values indicate significant *p*‐values.

^a^ Not significant in regression analysis.

Revision procedures were significantly associated with WHC occurrence, as demonstrated by both categorical and continuous analyses. In a chi‐square analysis, patients who underwent revision procedures had a markedly higher likelihood of developing WHC compared to those who did not (*p <* 0.001), corresponding to an odds ratio of 2.765. Furthermore, an independent samples *t*‐test revealed that the mean number of revision procedures was significantly greater among patients with WHC (mean difference = 0.468; WHC group range: 0–6 revisions vs. non‐WHC group: 0–3 revisions), with a 95% confidence interval of 0.3–0.8 (*p <* 0.001).

Primary closure was associated with a significantly lower risk of WHC development compared with secondary closure methods, such as negative pressure wound therapy (NPWT), skin grafts, and muscle flap reconstruction. The odds ratio of 0.228 (CI: 0.10–0.50; *p* < 0.001) indicates a 77.2% reduction in WHC likelihood with primary closure. Conversely, limb amputation showed no statistically significant association with WHC development (*p* = 0.108). Although infection was initially significant in the chi‐square analysis (*p* = 0.028), this significance disappeared in the multivariable regression model, suggesting it is not an independent predictor.

**FIGURE 1:**
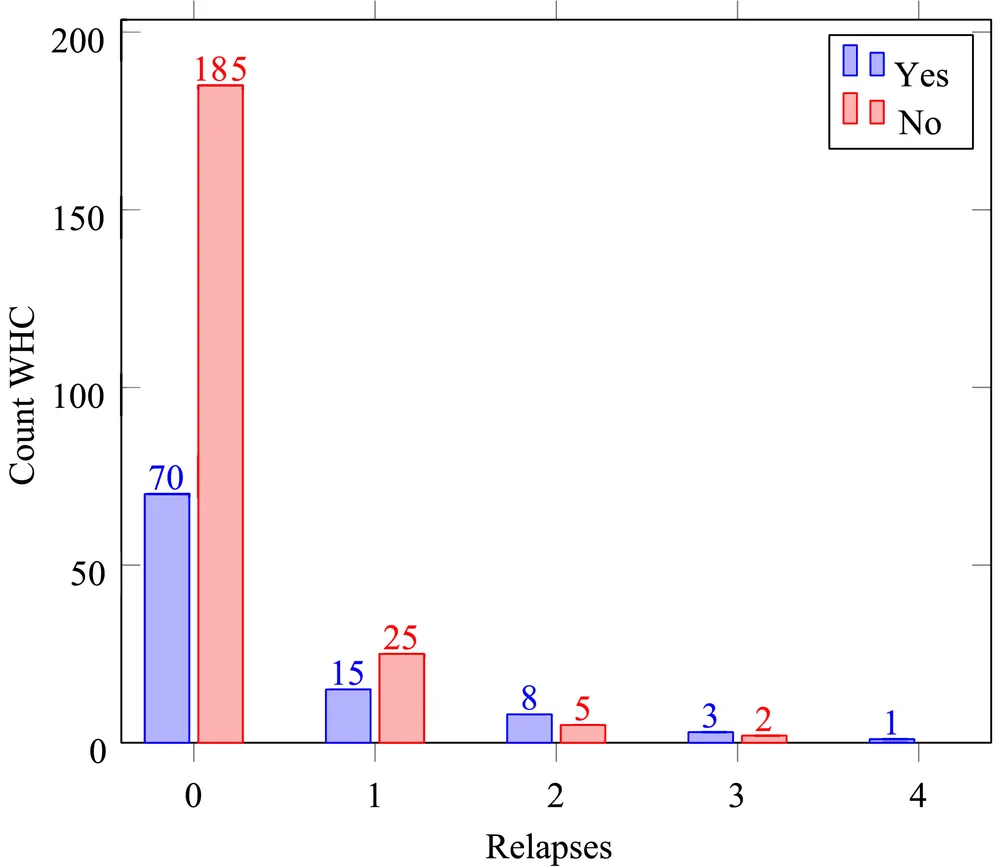
WHC development regarding number of relapses.

The relapse frequency among patients treated for STS was further analysed. Those with WHC experienced between 0 and 4 relapses, while patients without WHC had between 0 and 3. As shown in Table 2, the proportion of WHC cases increased steadily with the number of relapses (*p* = 0.012). An odds ratio of 0.575 (CI: 0.40–0.80) suggests a 42.5% higher risk of WHC with each additional relapse. This trend is also illustrated in the bar chart below (Figure 1). Interestingly, the R‐status was not significant in either univariate or multivariate analysis.

### 3.3 Multidisciplinary Variables

**FIGURE 2:**
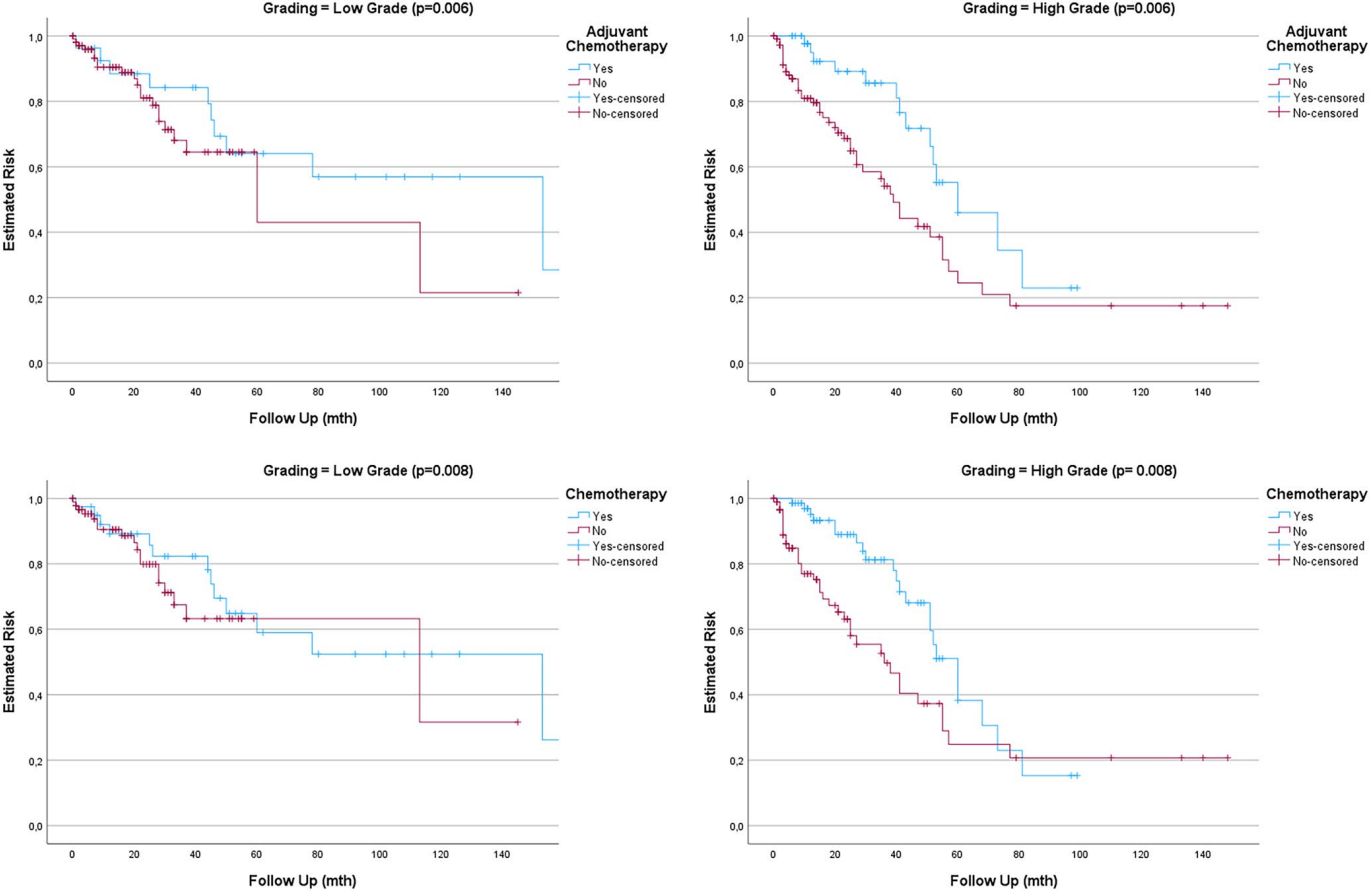
Estimated risk comparison for WHC development: chemotherapy versus adjuvant chemotherapy.

Predictive factors linked to modern multidisciplinary treatments, such as radiotherapy and chemotherapy, were analysed. No significant effect was found across the entire cohort. However, stratifying by initial tumour grade revealed subtle changes in treatment impact. In these subgroups, both chemotherapy and adjuvant chemotherapy showed a statistically significant protective effect. Radiotherapy had no significant impact, in either univariate or multivariate analyses, on the development of WHC. Kaplan–Meier curves indicated similar risk patterns for both treatments. It is important to emphasise that these results are based on small patient groups and should be interpreted cautiously. Overall, 34.6% (*n* = 110) of patients received chemotherapy, and 23.3% (*n* = 74) received adjuvant chemotherapy. When divided into low‐ or high‐grade groups, 71 of 173 (41%) high‐grade tumour patients and 39 of 145 (26.9%) low‐grade tumour patients received chemotherapy (Figure 2).

Analysis of quartile percentiles showed similar results across the treatment groups. Patients undergoing chemotherapy, regardless of whether it was neoadjuvant or adjuvant, had an estimated time to event of 84.25 months. Among these, patients with low‐grade tumours experienced a mean time to event of 118.85 months, while those with high‐grade tumours initially reached only 57.78 months.

In the subgroup receiving only adjuvant chemotherapy, the estimated time to event was 84.35 months. Notably, patients with low‐grade tumours experienced a mean interval of 124.95 months, while those with initially high‐grade tumours had a mean interval of 62.92 months.

Chemotherapy was primarily used for low‐grade pathologies including synovial sarcoma, liposarcoma, myxoid‐like sarcoma, and undifferentiated pleomorphic sarcoma. Two cases were classified as G3. All patients underwent neoadjuvant chemotherapy after biopsy confirmation. Adult patients were treated with Doxorubicin and Ifosfamide, while two paediatric cases were treated according to CWS protocol.

## 4 Discussion

The primary aim of this study was to identify independent and co‐independent negative predictive values (NPVs) for the development of wound‐healing complications (WHCs) in patients with soft tissue sarcomas (STS). The identified NPVs contribute to a high‐risk patient profile, which can influence future treatment strategies for STS. As noted in the introduction, established NPVs, such as age, tumour size, and location, have been shown to be significant risk factors for WHCs in STS treatment. However, variability among these factors presents ongoing challenges in contemporary STS management. Most of the literature has focused on multimodal treatment, particularly radiotherapy, rather than on the overall patient profile.

Expected risk factors such as size and localisation did not appear as NPVs in our study. Consistent with previous reports, tumours, particularly in the lower extremities, tend to be larger, measuring about 9–11 cm in diameter. As shown in Table 1, the most common tumour locations were in the lower extremities and pelvis, which could account for the larger average size [7, 9, 13]. Additionally, we could not link these factors to other variables in the multivariate analysis.

Consistent with Weitz et al., our analysis identified Metastasis as a significant NPV for WHC development [14]. Using our data, we were unable to determine whether there was a correlation with the disease's initial stage at diagnosis or with later disease progression due to relapse or incomplete resection (> R0). Disease progression is typically linked to immune system downregulation, which can be further influenced by various treatment modalities, as well as by increased oxidative stress, which impairs the body's healing capacity [15, 16]. Further studies have shown that reducing ROS levels with Negative‐Pressure Wound Therapy (NPWT) improves the overall outcome of surgical wounds in cancer treatment [16].

Initial wound‐closure strategies focused on three main techniques: direct wound closure, skin mesh grafts, and direct or delayed transplantation of free muscle flaps [2, 7]. In our study, secondary wound closures primarily used skin mesh grafts in combination with NPWT. Previous research has shown that NPWT provides substantial benefits in both acute and chronic wound management by promoting cellular proliferation, reducing edema, and stabilizing the wound environment by removing excess fluid [7, 17, 18, 19].

Our analysis revealed a strong correlation between the number of revision procedures (e.g., for margin control) and subsequent surgical interventions for recurrence, leading to an increased incidence of WHCs due to the increased surgical volume. Primary wound closure was predominantly performed, resulting in fewer WHCs.

In the literature, success rates for different techniques varied with tumour size. Smaller lesions were effectively managed with direct intraoperative wound closure, while larger defects showed excellent local control rates with free muscle flap techniques [2, 5, 20, 21]. For small‐volume tumours with significant skin involvement, skin mesh grafts were commonly used, although they were more susceptible to infection [2, 20, 21].

Advancements in STS treatment emphasise a multimodal approach that incorporates chemotherapy and radiotherapy. Current literature supports the significant impact of various treatment combinations on WHC outcomes. There is consensus that combining surgical intervention with radiation therapy enhances local disease control and improves overall survival [22, 23]. Most recent publications focus on radiotherapy and its relationship to WHC development, reporting WHC rates ranging from 20% to 40% [24]. In our dataset, we explored multiple approaches to multimodal therapy, acknowledging the potential limitation of small individual cohorts. We differentiated between the overall use of radiotherapy and its specific use as adjuvant or neoadjuvant treatment, along with tumour localisation, but found no impact on WHC development. Some studies have found no significant difference in local recurrence or survival between these treatment options. However, they observed a higher occurrence of WHCs in patients who had preoperative radiation and worse long‐term limb function in those treated postoperatively [25]. While research remains divided on whether multimodal therapy increases the risk of WHC, recent publications following standard time frames and concepts suggest it plays only a minor role.

Our research did not show any significant impact on the total population. However, when further divided by grading, we observed a positive predictive value for chemotherapy. Although not all STS types are sensitive to chemotherapy, we observed a relevant reduction in WHC risk in patients receiving neoadjuvant chemotherapy. As mentioned above, STS treated with chemotherapy show significantly lower WHC counts, as visualised in our Kaplan–Meier analysis. This opens a discussion about the importance of a proper evaluation of the multimodality of treatment and the consideration of chemotherapy to achieve potential early tumour size reduction and reduce the risk of late WHC development [26]. Notably, Hafsa et al. described an increased risk of WHC development in patients initially treated with radiation and later with chemotherapy [27]. However, due to reduced tissue supply after radiotherapy, further damage from chemotherapy cannot be adequately compensated for by the tissue. This could develop into a supporting stance for early chemotherapy intervention, with optional radiotherapy if required, but would require further large‐scale scientific investigation for proper evaluation.

In our study 8.5% of patients died during the follow‐up period, with 55.23% of these cases involving WHCs, implying an increased risk of an earlier death when affected by WHC. Combined with the significant finding of Infection associated with WHC, this can present as an increased risk of septic development and decreased immune function [28].

Additionally, we observed that patients with WHCs had a significantly longer initial length of stay than those without complications. A study by Wilke et al. reported that costs increased by over 20% with each return to the operating room. Given that WHCs often necessitate multiple re‐dressings, additional NPWT, or surgical reopening, the overall financial implications of these complications are considerable [29].

The limitations of our study include retrospective data collection. In a clinical study design, data could have been collected specifically for the question at hand. Further, due to the single‐institution design, we were not able to analyse different therapeutic approaches. We made this decision to minimise bias from treatment errors or delays and to ensure close follow‐up. We included only patients diagnosed and managed at our facility according to standardised protocols. A 2‐year interval was chosen to assess a manageable period without major changes in the therapeutic protocol. Follow‐up was also assessed after the set time interval.

## 5 Conclusion

Our findings suggest that patients with high initial tumour grading and repeated surgical procedures constitute a high‐risk group. Despite appearing clinically manageable at first, this profile carries a substantial risk of therapeutic error, reinforcing the importance of ongoing treatment reevaluation. Overall, the reduction of surgical intervention with the intent of primary wound closure should be considered for its potential to improve outcomes in this context. This proactive approach can help minimise the risk of WHC development, improve overall patient outcomes, and reduce overall costs.

## Funding

The authors have nothing to report.

## Ethics Statement

The research was approved by the Ethics Committee of the University of Duisburg‐Essen, reference number 24‐12119‐BO.

## Consent

All authors reviewed the manuscript and consented prior to submission.

## Conflicts of Interest

The authors declare no conflicts of interest.

## Data Availability

The datasets generated and/or analysed during the current study are not publicly available due to privacy regulations but are available from the corresponding author on reasonable request. All of the material is owned by the authors and/or no permissions are required.
